# Exploring Traditional Breast Cancer Risk Genes Among Aboriginal and Torres Strait Islander Women

**DOI:** 10.3390/life16081375

**Published:** 2026-08-20

**Authors:** Katie Meehan, Leanne Pilkington, Azim Khan, Nicholas Pachter, Lisa Spalding, Ayeisha Milligan Armstrong, Cameron Redfern, Andrew Redfern

**Affiliations:** 1 Harry Perkins Institute of Medical Research, 5 Robin Warren Dr., Murdoch, WA 6150, Australia; leanne.pilkington@health.wa.gov.au (L.P.); lisa.spalding@perkins.org.au (L.S.); ayeisha.milliganarmstrong@perkins.org.au (A.M.A.); cameron.redfern2020@outlook.com (C.R.); andrew.redfern@health.wa.gov.au (A.R.); 2 Centre for Medical Research, University of Western Australia, 35 Stirling Hwy., Crawley, WA 6009, Australia; nicholas.pachter@health.wa.gov.au; 3 Hollywood Private Hospital, 115 Monash Ave., Nedlands, WA 6009, Australia; azim.khan@health.wa.gov.au; 4 Western Australian Country Health Service, 189 Wellington St., Perth, WA 6000, Australia; 5 Genetic Health WA, Level 4 of Agnes Walsh House, 374 Bagot Rd., Subiaco, WA 6008, Australia; 6 Centre for Precision Health, Edith Cowan University, 270 Joondalup Dr., Joondalup, WA 6027, Australia; 7 School of Diagnostic and Therapeutic Sciences, Curtin University, Kent St., Bentley, WA 6102, Australia; 8 Fiona Stanley Hospital, 11 Robin Warren Dr., Murdoch, WA 6150, Australia

**Keywords:** Aboriginal Australians, breast cancer, *BRCA*1/2, pathogenic variants, family history

## Abstract

Background: Germline pathogenic variants in *BRCA*1/2 increase breast and ovarian cancer risk, but their prevalence in Aboriginal and Torres Strait Islander (herein referred to as Aboriginal) families has never been studied. Consequently, their contribution to breast cancer in this population is unknown. Methods: This retrospective cohort study included 259 Aboriginal women and 789 age- and remoteness-matched non-Aboriginal women diagnosed with breast cancer (2001–2016). We assessed family history, genetic service referral and testing rates, and pathogenic variant rates in the context of testing access. Results: Family history data were available for more Aboriginal than non-Aboriginal cases (46% vs. 34%, *p* < 0.001), with no difference in reported first-degree relatives with breast (22.5% vs. 22.4%, *p* = 0.98) or ovarian cancer (4.2% vs. 2.2%, *p* = 0.29). Similar proportions were referred to genetic services for testing eligibility (5.4% vs. 7.2%, *p* = 0.392). Of those referred, similar proportions were offered testing (86% vs. 81%, *p* = 0.66). Across the full cohort, *BRCA*1/2 pathogenic variants were detected in 0.77% of Aboriginal versus 2.4% of non-Aboriginal women (*p* = 0.127); any pathogenic gene variant was detected in 0.77% versus 3.0% (*p* = 0.039). Conclusions: Pathogenic genetic variants were detected less frequently in Aboriginal women, although, in keeping with international clinical practice, most women diagnosed with breast cancer were not tested. Given that only a small proportion of the cohort underwent germline testing, this study cannot distinguish whether the lower detection rate reflects a genuinely lower population level prevalence of pathogenic variants or differences in historical referral, selection, and testing practices. These findings are hypothesis-generating and require validation in larger Australian Aboriginal studies including unselected populations with whole-population testing data.

## 1. Introduction

National Australian statistics consistently show that Aboriginal women with breast cancer have sub-optimal outcomes relative to non-Aboriginal people [1]. Although modern medical practices have brought advantages to general populations, Aboriginal Australians have not received equitable benefit. Evidence consistently shows that Aboriginal people’s health outcomes have been and continue to be negatively impacted by colonisation and racism with attendant cultural disruption and socioeconomic disparity. The widening cancer mortality gap associated with breast cancer is undoubtedly associated with the numerous barriers Aboriginal people may experience throughout the cancer care continuum [2]. In contrast, Aboriginal populations have frequently been observed to have lower incidences of breast cancer [1].

A range of well-established risk factors that modulate breast cancer risk include demographics, lifestyle, reproductive history, and hormone levels [3]. Inherited genetic factors are also important [4]. Females carrying a variant in *BRCA*1 (BReast CAncer gene 1) or *BRCA*2 (BReast CAncer gene 2) genes are at high risk of developing early-onset breast and ovarian cancer [5,6,7]. International data based on ‘general’ populations quantify these risks for carriers of *BRCA*1 or *BRCA*2 pathogenic variants for developing breast cancer at ~65% and ~45% respectively and for ovarian cancer at ~39% and ~11% by the age of 70 [8,9].

Pathogenic *BRCA*1 and *BRCA*2 variants are rare (estimated at 0.4–0.5% of the whole population) and are responsible for only ~5% of all breast cancers [10,11] although calculating prevalence accurately is difficult due to the rarity of whole-population-based screening initiatives. The proteins encoded by *BRCA*1 and *BRCA*2 risk genes are essential components in the homologous recombination pathway that is responsible for repairing double-stranded DNA breaks to avoid pathogenic consequences. An expanding range of pathogenic variants in other genes that confer an increased breast cancer risk have been identified including *PALB2*, *TP53*, and *PTEN*. Assays for these have been added incrementally to the standard panel of genetic changes studied [9]. Pathogenic variants in these genes are usually inherited in an autosomal dominant Mendelian manner, whereby an offspring who inherits one defective and one functional copy of the gene from each parent is at an increased risk of breast cancer. The functional gene copy is usually initially sufficient to produce enough protein product to service homologous repair pathways. However, incremental DNA damage over time due to increasing age, lifestyle factors, and environmental exposures, in concert with mutation or the silencing of the functional copy, leads to DNA damage accumulation and subsequent cancer development.

Pathogenic variant carriage rates have been compared between some populations of differing ancestries. Relatively large studies are available for African American women. A recent large study found no significant difference in germline pathogenic variant prevalence rates between Black (5.6%) and non-Hispanic White women (5.1%; *p* = 0.12) [12]. Similar overall carriage frequencies are also reported between Chinese and White women, although rates of individual gene changes varied [13].

Information on smaller Indigenous populations is lacking. Data on New Zealand Māori are sparse with no confirmed pathogenic variants found in 23 unselected Māori patients undergoing testing from a general New Zealand population [14]. No larger studies were identified. No case series were found for native North American people, although an individual family carrying a *BRCA1* gene has been reported [15]. Despite the well-established lower breast cancer incidence in Aboriginal women, no studies have explored the relative carriage rates of pathogenic variants in breast cancer risk genes in this population.

Therefore, the aim of this study was to investigate the prevalence of family history, genetic testing rates, and carriage of pathogenic variants of breast cancer risk genes among a substantial matched cohort of Aboriginal and non-Aboriginal people diagnosed with breast cancer in Western Australia.

## 2. Materials and Methods

Positionality. The research team comprised Aboriginal and non-Aboriginal people with varied lived experience in the health and research sectors. As a collaborative team, we have worked to ensure the research is not deficit-driven and is conducted in a way that will ensure Aboriginal people will benefit.

Intent. To compare prevalence of family history of breast and ovarian cancer, we studied referral rates for genetic testing and rate of carriage of pathogenic variants of established breast cancer risk genes between Aboriginal and Torres Strait Islander and non-Aboriginal Western Australian women.

Cohort Selection. We retrospectively identified a cohort of 259 Aboriginal Australians diagnosed with breast cancer between 2001 and 2016. This represents all women reported to the Western Australian Cancer Registry (WACR) diagnosed with breast cancer who self-identified as Aboriginal in that time period. Each Aboriginal participant was matched within the registry by age and remoteness with three non-Aboriginal participants diagnosed across the same period. Remoteness of residence was measured using the Accessibility/Remoteness Index of Australia (ARIA) and socioeconomic categories were assessed using the Index of Relative Socioeconomic Disadvantage (IRSD) score. Both were calculated from residential postcode at the time of diagnosis and assigned to each patient. ARIA 5 comprises five categories including major cities (highly accessible), inner regional (accessible), outer regional (moderately accessible), remote (poorly accessible), and very remote (very poorly accessible). ARIA 3 combines categories and is limited to major cities (highly accessible), regional (moderately accessible), and remote (poorly accessible).

Genomic Testing. Western Australia’s clinical genetic services began offering *BRCA* testing in 2001, initially for high-risk individuals with strong family histories, which broadly coincided with the cohort start date of 2001. Testing at this time included the entire coding sequences and each intron/exon boundary of *BRCA*1 and *BRCA*2 to identify variants, duplications, and deletions. From 2001 to 2016, *BRCA* testing evolved from limited, targeted approaches to more accessible, technology-driven services. The introduction of next-generation sequencing and Medicare funding in 2017 were major milestones that expanded access and integrated testing into routine cancer care. Testing was expanded in 2016 to include select exons of *ATM* and *CHEK2* and then again in 2017 with inclusion of the TruSight Breast Cancer gene panel that assays *BRCA*1, *BRCA*2, *TP53*, *CDH1*, *CHEK2,* and *PTEN* variants as well as *PALB2* and *ATM* truncating variants.

Knowledge Gathering. Basic tumour staging parameters as well as invasive tumour grade (modified Bloom and Richardson grading system [16] and receptor expressions all assessed by immunohistochemistry) were obtained from the WACR database. When data were incomplete, additional data were extracted from routine pathology assessment reports and by contacting pathology service providers. When standard pathological tests had not initially been performed, extra tissue sections (when available) were scored for missing values. Receptor positivity was defined as ≥1% nuclear staining by immunohistochemistry for both oestrogen receptor (ER) and progesterone receptor (PR). HER2 positivity was defined as IHC 3+ or IHC 2+ with confirmatory in situ hybridisation amplification. Triple negative breast cancer was defined as ER negative, PR negative, and HER2 negative by these criteria.

Regarding family history, data on relatives in the family with breast, ovarian, and other cancers were collated from available medical records, including whether cases were in first degree, second degree, or more distant relatives. To map the genetic service referral process in the study cohort, information on genetic services referrals offered and made, as well as information on germline pathogenic variant tests offered and conducted were also extracted from hardcopy and digital medical records. Cases in which medical records stated that a genetic services referral was either being made or under consideration, but which lacked results were cross-checked with the state Familial Cancer Program records.

Medical Actions Guided by *BRCA* Pathogenic Variant Carriers. The identification of germline *BRCA*1/2 pathogenic variants can lead to alterations in or additions to standard management. We also sought evidence from the WACR data, additional pathology reports, and patient hospital medical records of initial surgery type (local excision vs. mastectomy), adjuvant chemotherapy delivery (inclusive of the use of platinum-based agents), use of Poly (ADP-ribose) polymerase (PARP) inhibitors, second primary tumours (ovarian or breast), and prophylactic surgeries (ipsilateral or contralateral mastectomy and oophorectomy). This part of the study was confined to *BRCA*1/2 pathogenic variant carriers, as risk levels and appropriate actions for pathogenic variants in other genes are not yet established.

Statistical Analysis. Chi-square testing was used to determine associations between categorical variables (tumour demographics, *BRCA*1/2 status, family history, and Aboriginal status). Fisher’s exact test was used in cases of sparse data. Age ranges, Ki67, and other continuous data were compared using two tailed *t*-tests. Multivariable logistic regression was performed to assess associations between Aboriginal status and family history documentation, referral for genetic evaluation, and acceptance of genetic testing, adjusted for socioeconomic disadvantage (IRSD decile) and tumour grade. Geographic remoteness was not included as a covariate as this was a matching variable in the study design. Germline pathogenic variant status was not modelled, as only 2 and 24 pathogenic variants were identified in the Aboriginal and non-Aboriginal cohorts, respectively. Firth’s penalized logistic regression was used for the acceptance-of-testing model given the small number of observations with a recorded testing decision. Significance was determined based on *p* < 0.05. Statistical analyses were performed using SPSS (version 29.0.0.0(241), IBM) and R (version 4.5.2 (2025-10-31 ucrt), packages logistf, broom).

Study Ethics and Governance. Initial guidance and oversight for the research design and conduct were kindly provided by the Aboriginal Reference Group of BreastScreen WA. Ongoing guidance was subsequently provided by the WA Aboriginal Cancer Collaborative. Currently, the work is led, guided, and safeguarded by the Aboriginal Research Team Against Cancer (ARTAC), a group of Aboriginal people who have a lived experience of cancer or have cared for loved ones with a lived experience of cancer.

Our research was conducted in accordance with the National Health and Medical Research Council’s guidelines for conducting ethical research with Aboriginal and Torres Strait Islander peoples [17]. This study was reviewed and approved by the WA Aboriginal Human Ethics Committee (WAAHEC) (HREC838) to ensure all ethical guidelines were met and that governance was controlled by Aboriginal people as per the “Consolidated criteria for strengthening reporting of health research involving Indigenous peoples” statement. The study was also approved by the WA Department of Health (Project 2013/51), the South Metropolitan Health Service (RGS0451), and reciprocal approval was obtained from the University of Western Australia (RA/4/20/4101). This manuscript was also reviewed prior to publication by WAAHEC and the WA Department of Health.

## 3. Results

### 3.1. Patient and Tumour Demographics

The study population consisted of 259 Aboriginal and 789 non-Aboriginal breast cancer cases (Table 1). All cases in this study were female. The average age and age range of breast cancer diagnosis among Aboriginal (56 years) and non-Aboriginal (55 years) women were not statistically different (*p* = 0.858), consistent with the age-matched design of the study. There was also no significant difference for year of diagnosis between the Aboriginal and non-Aboriginal cohorts (*p* = 0.510). A significant difference was observed for ARIA5 matching (*p* < 0.0001) but not ARIA3 (*p* = 0.495). This arose due to an insufficient number of non-Aboriginal women being diagnosed with breast cancer who were living very remotely. Additional non-Aboriginal women living in remote areas were included to achieve the best match available. Considering socioeconomic disadvantages, substantial differences in IRSD were observed (*p* < 0.001) with 26% more Aboriginal patients included in the most disadvantaged category and a similar excess of non-Aboriginal patients coming into the two categories with the least disadvantage.

Considering tumour differences, staging was higher in Aboriginal women who were diagnosed with larger tumours (*p* < 0.001) but with similar distant (*p* = 0.065, OR 1.69, 95% CI 0.93–3.02) and lymph node metastases (39.9 vs. 34.7%, *p* = 0.158, OR 1.25, 95% CI 0.91–1.71) (Table 2). Significantly more frequent adverse biological features were also seen for the Aboriginal cohort with a higher proportion of Grade III disease (42.5 vs. 29.7%, *p* < 0.001, OR 1.79, 95% CI 1.32–2.43), less frequent oestrogen receptor (71.8 vs. 77.2, *p* = 0.016, OR 1.49, 95% CI 1.05–2.10) and progesterone receptor (64.1 vs. 69.7%, *p* = 0.027, OR 1.42, 95% CI 1.03–1.95) positivity, and greater HER2 expression (21.3 vs. 13.3%, *p* = 0.004, OR 1.76, 95% CI 1.18–2.59), respectively.

### 3.2. Family

Family history data were available for 120 of 259 Aboriginal women (46.3%) and 268 of 789 non-Aboriginal women (34.0%), with significantly more Aboriginal patients having available data (unadjusted *p* < 0.001, aOR 1.63, 95% CI 1.21–2.20, adjusted *p* = 0.0014). All subsequent family history analyses are reported among patients with available data only (Aboriginal *n* = 120, non-Aboriginal *n* = 268), unless otherwise stated.

Among those with family history data available, the frequency of any family history of breast or ovarian cancer did not differ significantly between Aboriginal and non-Aboriginal women (44.2% (53/120) vs. 51.5% (138/268), *p* = 0.18). Similarly, there were no significant differences in the rates of first-degree relatives with breast cancer (22.5% (27/120) vs. 22.4% (60/268), *p* = 0.98) or ovarian cancer (4.2% (5/120) vs. 2.2% (6/268), *p* = 0.29). Endometrial cancer was noted given its association with *BRCA*2 pathogenic variant carriage, but was rarely observed, occurring in fewer than 1% of first- or second-degree relatives in either cohort (Table 3).

### 3.3. Genetic Testing Rates for Germline Cancer Risk Pathogenic Variants

No significant differences in rates of genetic testing offered or accepted were found, although the low numbers precluded the validation of modest differences. There was no difference in the proportion of the Aboriginal and non-Aboriginal cohorts who were referred to state genetic services or who were assessed by that service for germline testing (5.4 vs. 7.2%, respectively, *p* = 0.392, aOR 0.94, 95% CI 0.55–1.56). Similarly, there was no statistical difference in the proportion of Aboriginal and non-Aboriginal cohorts that were offered testing once assessed (12/14 (86%) vs. 46/57 (81%), *p* = 0.66). Grade 3 disease was independently associated with higher odds of referral, regardless of Aboriginal status (aOR 2.81, 95% CI 1.46–5.87, *p* = 0.003). Finally, acceptance of testing was similar among both groups (10/12 (83%) vs. 44/46 (96%), *p* = 0.13, aOR 0.48, 95% CI 0.04–6.53) (Table 4). The genetic results for two individuals recorded as tested in the non-Aboriginal cohort were not available as these were carried out by private genetic testing providers and so were not available through genetic services records. There were no significant differences in ARIA3, ARIA5, or IRSD between Aboriginal and non-Aboriginal people who were assessed, offered testing, or accepted/declined testing.

### 3.4. Comparing Pathogenic Variant Rates Across the Complete Cohorts

Considering the full cohort, there was no significant difference in the number of Aboriginal patients relative to non-Aboriginal patients with *BRCA*1 and/or *BRCA*2 pathogenic variants (2/259 vs. 19/789 cases, *p* = 0.127). Across all risk genes tested, pathogenic variants were detected less frequently in Aboriginal patients (2/259 vs. 24/789, *p* = 0.039, OR 0.25 (95% CI 0.028–1.01)). The accuracy of this estimate is limited by the small number of events and is best interpreted as hypothesis-generating rather than a stable effect estimate.

**Figure 1:**
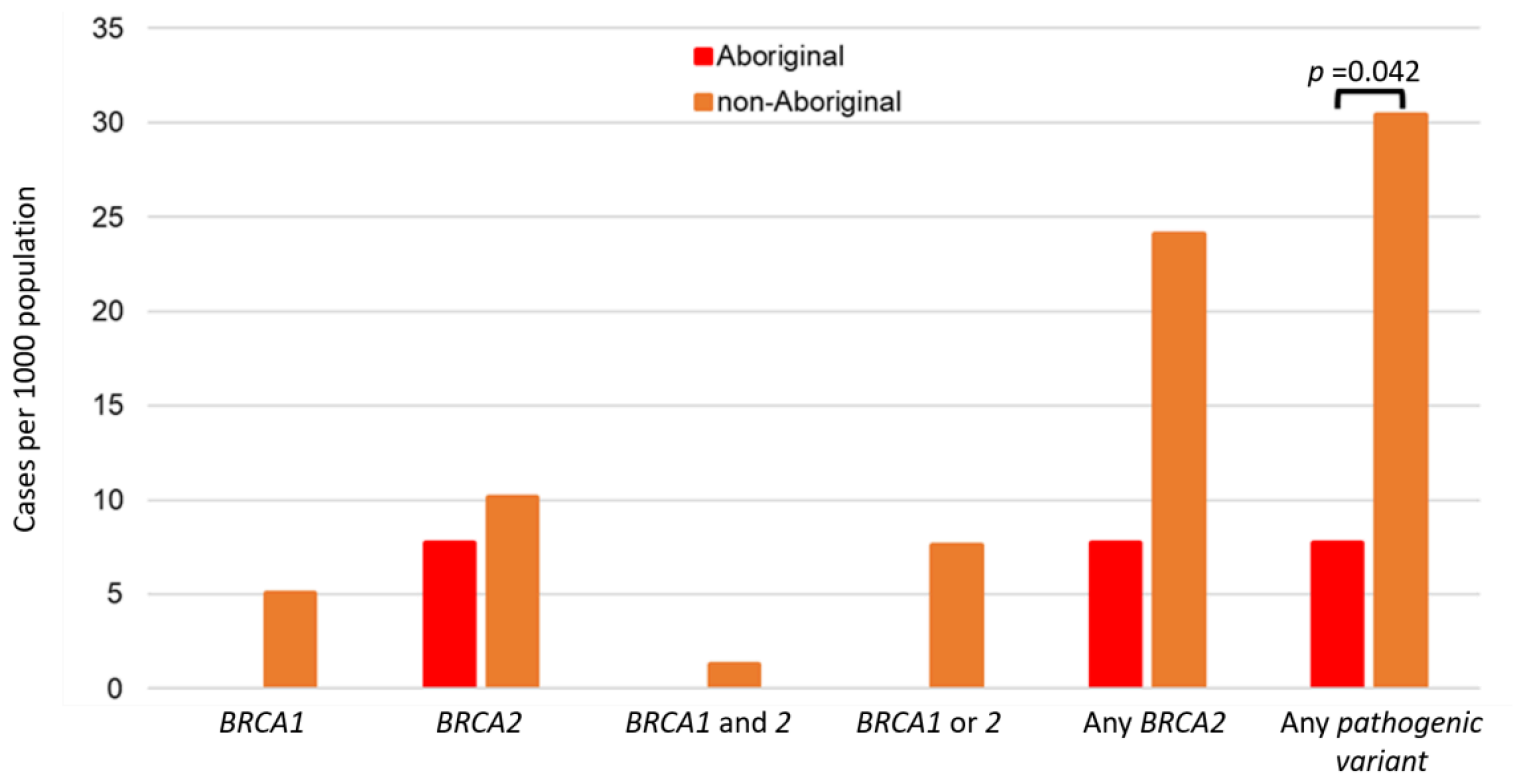
Detected pathogenic germline variants per 1000 cohort members. Given that majority of cohort members were not genetically tested, variant rates per 1000 population were calculated by dividing the number of positive cases by the population included in the total study cohort (rather than tested cohort) and multiplied by 1000.

Crude rates of pathogenic variants identified per 1000 patients are shown in Figure 1. For any pathogenic *BRCA* variant, rates in Aboriginal and non-Aboriginal cohorts were 7.7 cases and 24.1 cases per 1000 patients, respectively (*p* = 0.127). Considering rates of the identification of pathogenic variants for any cancer risk gene, the corresponding rates were 7.7 and 30.4 cases per 1000, with the difference reaching statistical significance (*p* = 0.039). 

Looking at the specific variants involved, pathogenic *BRCA*2 variants were found in two Aboriginal breast cancer cases. No pathogenic *BRCA*1 variants or variants in any other cancer risk genes tested were identified in Aboriginal patients, giving a positivity rate for any *BRCA* pathogenic variant of 20% (2/10) in the Aboriginal patients tested (Table 4).

For the non-Aboriginal cohort, four pathogenic *BRCA*1 variants, eight pathogenic *BRCA*2 variants, one combined pathogenic *BRCA*1 and *BRCA*2 variants, and six pathogenic *BRCA* variants where specific gene (*BRCA*1 or *BRCA*2) was not recorded were identified. Further single cases of pathogenic variants in *BARD1*, *CHEK2*, *TP53* (Li-Fraumeni syndrome), and *STK11* (Peutz-Jeghers syndrome) were found. Specific information regarding the variant type was not available for one additional case. This gave a positivity rate for any *BRCA* pathogenic variant in those tested of 43% (19/44) and a positivity rate for any risk gene pathogenic variant of 54% (24/44).

### 3.5. Comparing Pathogenic Variant Rates in the Tested Populations

When comparing positivity rates in the women tested, the Aboriginal women showed a numerically lower rate of any pathogenic variant compared with the non-Aboriginal women (2/10 (20%) vs. 24/44 (54.5%), *p* = 0.079, OR 0.21 (95% CI 0.020–1.24)), though this did not reach statistical significance, possibly reflecting the small number of Aboriginal women tested. The positivity rate for *BRCA* variants in those tested was also lower in the Aboriginal compared with non-Aboriginal women, though again this did not reach statistical significance (2/10 vs. 19/44, respectively, *p* = 0.284).

### 3.6. Action Taken Due to Identified Pathogenic Variants

Identification of a pathogenic variant in a DNA repair gene, particularly one of the two *BRCA* genes, may lead to the consideration of changes or additions to management. As outlined in the methods section, data on these interventions were sought. We also looked for evidence of additional incident breast and ovarian cancers and their management.

Low numbers of *BRCA* pathogenic variant carriers in the Aboriginal population precluded useful comparisons between cohorts. Additionally, both Aboriginal patients and most non-Aboriginal patients were treated prior to the routine consideration of platinum addition to adjuvant chemotherapy regimens and before the trialling and subsequent approval of PARP inhibitor therapy [18]. Both Aboriginal patients had second contralateral primary breast cancer some years after their initial primary tumour. One had a lumpectomy for the initial primary and then opted for a bilateral mastectomy at the time of the contralateral diagnosis. The second patient opted for mastectomies at the time of the two separate diagnoses. Both patients underwent prophylactic oophorectomy, and both received adjuvant chemotherapy, although neither received platinum agents nor PARP inhibitors for either diagnosis.

Turning to the non-Aboriginal patients, three had bilateral primaries at diagnosis and two went on to develop contralateral primaries at later dates. Additionally, ovarian and fallopian tube carcinomas were diagnosed in two patients with metachronous and synchronous bilateral breast carcinomas, respectively. Mastectomy was the initial surgery chosen in 12/19 patients (63%) with a further therapeutic or prophylactic ipsilateral mastectomy occurring in five additional patients. On the contralateral side, five further patients required surgery either at the time of initial diagnosis or a later date for contralateral cancers and 11 opted for prophylactic contralateral mastectomies. Oophorectomy was performed therapeutically and prophylactically in two and ten patients, respectively, leaving seven patients with intact ovaries. Again, no non-Aboriginal patients received platinum-based adjuvant chemotherapy or a PARP inhibitor for breast cancer, although one did receive the latter for a subsequent fallopian tube carcinoma.

## 4. Discussion

The identification of familial pathogenic variants in genes such as *BRCA*1 or *BRCA*2 in newly diagnosed breast cancers has important and expanding treatment implications, guiding future cancer prevention strategies, directing both local and systemic therapy for diagnosed cancers, and facilitating the risk management of relatives. This is the first study examining testing and detection rates of germline pathogenic variants in breast cancer risk genes in Aboriginal women. Among the small proportion of women who underwent genetic testing, pathogenic variants were detected 2.7-fold less frequently in Aboriginal women than non-Aboriginal women (20% vs. 54.5%, *p* = 0.079, OR 0.21, 95% CI 0.020–1.24), although this was not statistically significant, possibly reflecting the very small numbers tested. Across the full cohort, the majority of whom were not tested, any pathogenic variant was detected 3.9-fold less frequently in Aboriginal women (0.77% vs. 3.0%, *p* = 0.039, OR 0.25, 95% CI 0.028–1.01). This estimate is also imprecise given the small number of events and is best regarded as hypothesis-generating. No *BRCA*1 pathogenic variants were detected among the 10 Aboriginal women who underwent testing. This is consistent with the national findings that Aboriginal women are less likely to be diagnosed with breast cancer, although one would not anticipate that germline pathogenic variant differences alone could account for this, given that only around 5% of breast cancers occurring in general populations are due to pathogenic variants in these known risk genes [10,18]. In addition to the low rate of pathogenic variants in the Aboriginal cohort, it is important to note that we do not have detailed lineage data on these cases such that it is unknown whether the pathogenic *BRCA*2 variants identified originated from Aboriginal or non-Aboriginal ancestors.

Considering explanations for this lower observed pathogenic variant carriage rate, this may be an artifact of low case numbers and population testing confounders or may accurately reflect a lower carriage in Aboriginal people.

Considering first confounding factors, *BRCA*1/2 pathogenic variant data are largely derived from European and Caucasian populations, and it is unknown whether this is broadly applicable to or useful for Aboriginal Australians [19]. The prevalence of *BRCA*1 and *BRCA*2 pathogenic variants differs extensively among diverse ethnic groups [20,21], as do the specific variants carried by ancestry, with 84% of *BRCA*1 and 80% of *BRCA*2 pathogenic variants shown to be confined to a single ancestral population [22]. As the geographic distance from Europe increases, the private variant rate also rises with 44.2%, 51.2%, 54.2%, and 88.9% of *BRCA* pathogenic variants being private to Indian, Chinese, Korean, and Japanese populations relative to European populations [23]. Another example comes from studies showing that two widely used Western *BRCA* pathogenic variant prediction models (*BRCA*PRO [24] and Myriad II [25]) underestimate the risk of *BRCA*1/2 pathogenic variants in Asians [26] and Koreans [27]. Consequently, our study may show a falsely low pathogenic variant rate due to the testing panels applied. Studies specific to Australian Aboriginal peoples could reveal *BRCA* pathogenic variants unique to this population, allowing for accurate population-specific screening for risk alleles and the optimization of guidance for prevention, screening, and management.

Additionally, the data gathered reflect the results of standard clinical practice, where the family history and cancer subtype of newly diagnosed breast cancer patients are considered and, if certain risk thresholds are met, referral to genetic services and genetic testing should take place. Consequently, although the rules for access to testing were identical for the two populations, the majority of both populations were not tested, introducing the potential for bias in selecting testing candidates. Notably, testing thresholds have fallen over time, reflecting our expanding knowledge of risk and the falling costs of testing. Consequently, repeating our study for a more contemporary population should result in higher testing rates, making statistically valid comparisons more feasible.

Relating to this, it is increasingly recognized that some germline pathogenic variant carriers with breast cancer may have little or no family history, leading to a hidden burden of undetected pathogenic variants. Demonstrating this, in a contemporary Australian population of 429 women with breast cancer, the rate of pathogenic germline variant carriage in those meeting testing eligibility criteria was 3.0% (13/429), identical to that seen in our non-Aboriginal cohort. However, a further 3.5% of participants who did not meet testing criteria also harboured pathogenic variants, resulting in a rate of 6.5% in the full unselected population [28]. It is possible that our Aboriginal population could have a higher pathogenic variant rate than their non-Aboriginal peers in testing-ineligible patients.

Hypothetically, a founder effect could also produce a genuinely lower carriage rate if the founding population from which Aboriginal Australians descend carried few pathogenic risk alleles. In addition, genetic drift and geographic isolation over subsequent millennia could sustain a lower population-wide prevalence. It has also been proposed that high genomic variability in *BRCA1*/2 persists across populations because it confers a survival advantage against evolving pathogens [29], with archaeogenetic data suggesting most known pathogenic variants arose within the last 5000 years [30,31], after the isolation of the Aboriginal population [32]. Whether differing pathogen exposure over this period could plausibly have favoured genomic stability over variability in the Aboriginal population, and thereby produced a lower variant carriage rate, is an interesting question but remains entirely untested by the current data and would require exploration via dedicated population genomic studies.

Interestingly, we did not find a lower prevalence of family history in the Aboriginal group with equally prevalent family histories, despite the known lower incidence of breast cancer. This extended across breast and ovarian cancer and by degree of relative. Globally, 15–30% of women with breast cancer report at least one first-degree relative with the disease [33,34]. An Australian study contemporaneous with our retrospective population found that 38% (*n* = 24) of Aboriginal patients with breast cancer had a family history of the disease [31] and 22% (*n* = 14) had an affected first-degree relative, aligning closely with our figures. We note that, as per the most recent Australian census [35], the average Aboriginal family is larger than the average non-Aboriginal family. This could result in a higher number of first- and second-degree relatives for Aboriginal patients which could increase family case numbers relative to non-Aboriginal peers in the absence of an actual increase in risk.

The analysis of family history is confounded by significant missing data in both groups, potentially due to the retrospective nature of the study, where a negative family history in medical records may not have been recorded, reflecting a recording bias by medical practitioners. Alternatively, a greater proportion of Aboriginal patients could be aware of their family history, be more likely to volunteer family history information, or medical practitioners may be more likely to ask Aboriginal patients about their family history. The difference in data availability persisted after adjustment for socioeconomic disadvantage and tumour grade (aOR 1.63, 95% CI 1.21–2.20, *p* = 0.0014) and was stable by year of diagnosis, indicating it is not explained by these factors. A prospective study incorporating a detailed compilation of pedigrees would be required to circumvent these factors.

We report that the rates of genetic service referrals and offers for testing were not significantly different for Aboriginal and non-Aboriginal patients. We also did not observe a significantly lower uptake of testing by patients once offered, with 83% of Aboriginal people accepting genetic testing. These findings suggest that lower detected variant rates are not solely attributable to lower rates of testing being offered or accepted, although the small numbers involved mean this cannot be excluded. The adjusted analysis revealed that tumour grade was independently associated with referral for genetic evaluation, with Grade 3 disease associated with over twice the odds of referral (aOR 2.81, 95% CI 1.46–5.87, *p* = 0.003). This is likely due to the association between high grade and triple negative disease, rather than any disparity in referral practice by Aboriginal status.

In the USA, in a high-risk population, Black patients were over five times less likely to have genetic testing than non-Hispanic White patients after controlling for appropriate variables [36]. This is despite the confirmed utility of standard testing panels for identifying high-risk individuals in the African American population [37]. Acknowledging the low case numbers, this stark difference was not observed in our matched populations despite concerns expressed in the literature associated with genetic data privacy, sovereignty, and broader risks to the community in Aboriginal populations. A large Queensland study exploring perception of familial risk in 252 Aboriginal patients found that 68% of participants had concerns about their relatives being affected by cancer and 54% indicated they would like a specialist assessment of their familial risk [31]. A qualitative study of genetic counsellors and clinical geneticists with experience counselling and informing Aboriginal clients identified that being flexible and practical, taking time to build rapport, and recognizing different family structures and decision-making processes, as well as socioeconomic disadvantage, were important factors when interacting with Aboriginal clients [38].

In the context of this willingness to undergo genetic testing in the WA Aboriginal community, failing to understand the ancestry-specific genomic underpinnings of disease and risk due to failure to establish broad testing programs poses a threat to the well-being of people in Aboriginal communities [39]. Specific genomic studies in Aboriginal populations are required to avoid this but need careful and considered design with both representation from the communities from which participants will be drawn as well as from clinical geneticists regarding the potential benefits the participants may experience.

In this regard, all research involving Aboriginal people should be led by or co-designed with Aboriginal people, a model built into this research upon following the Australian Institute of Aboriginal and Torres Strait Islander Studies Code of Ethics [40] as a way of recognising and respecting Aboriginal and Torres Strait Islander people’s right to self-determination. Retrospective studies, as described here, draw on a wealth of existing data but rely on a waiver of consent [41]. To ensure that the rights of Australian Aboriginal people whose data are involved in research are acknowledged and protected, extensive consultations with Aboriginal people are crucial. For the current study, the collective research data are owned and controlled by Aboriginal team members as per the Maiam nayri Wingara Indigenous Data Sovereignty Principles and observing the CARE Principles (Collective benefit, Authority to control, Responsibility, Ethics) [42] and FAIR (Findable, Accessible, Interoperable, Reusable) data standards [43].

Of interest, there are recent efforts exploring the need and feasibility of universal, routine germline testing in the whole breast cancer population [44,45], particularly with the expanding array of specific interventions tailored to pathogenic variant carriers described below. It has been shown that universal testing for pathogenic variant carriage in willing participants after a breast cancer diagnosis can lead to a breakdown of barriers to testing, identification of additional cases with pathogenic variants but lacking substantive family history, revision of treatment plans, and increased availability of care for family members at risk [46]. Such a universal genetic testing program including Aboriginal people should have powerful and practice-changing outcomes. Appropriately, discussions around acceptance of genetic testing among Aboriginal Australians have been recently reignited [44,45].

Tailoring of breast cancer management in pathogenic variant carriers includes advising intensified cancer screening [47] and pharmaceutical prophylaxis [48,49] in patients and affected family members. *BRCA*-associated hereditary breast cancers are more susceptible to DNA-damaging chemotherapies such as cisplatin [50] and PARP inhibitors [51], with the latter now standard of care in the adjuvant treatment of higher-risk *BRCA*-associated breast cancers [51] as well as in metastatic disease [19] in Australia. Our assessment of treatment delivered to those with pathogenic *BRCA* variants was limited by the small number of Aboriginal people affected as well as by the fact that both were diagnosed two decades ago, since which time practice has evolved. However, both Aboriginal patients underwent eventual bilateral mastectomies and adjuvant chemotherapy and had prophylactic oophorectomies. In the non-Aboriginal patients, 89% underwent ipsilateral mastectomy, 79% opted for contralateral mastectomy, and 63% had bilateral oophorectomy. No patients received adjuvant platinum or PARP inhibitors due to the recent addition of these interventions to care.

*Limitations*. Despite being the first exploration of its kind, this study has clear limitations. First, this is a retrospective study with limited numbers. While we sought to include all Aboriginal breast cancer cases diagnosed in Western Australia between 2001 and 2016 (*n* = 259), results for *BRCA*1/2 testing were available for only a small proportion of cases, largely reflecting the historical practice of focused genetic testing only in high-risk individuals. The slightly lower rate of testing in the Aboriginal cohort may be commensurate with the lower incidence of breast cancer among Aboriginal Australians, although we cannot exclude bias or undefined practical limitations associated with clinical care.

Eligibility for *BRCA*1/2 testing includes a diagnosis with breast or ovarian cancer or detection of a pathogenic variant in a family member. However, if a person is diagnosed with breast cancer or has a *BRCA*1/2 pathogenic variant detected, the choice of an individual to disclose this information to their relatives could vary by Aboriginality, which could have impacts on risk assessment and consequently on referral rates. Considering the overall low numbers of cases that were tested by genetic services, under-testing may have occurred and might confound the conclusions that have been made about the true prevalence of the pathogenic variants.

Additionally, as with all cancer registries, the WACR relies on self-identification of Aboriginal status to classify affected individuals. Consequently, reluctance amongst Aboriginal people to self-identify through fear of discrimination could introduce bias and impact the results.

## 5. Conclusions

Overall, the results suggest that a family history of breast or ovarian cancer is equally common among Aboriginal and non-Aboriginal Western Australians affected by breast cancer. In contrast, pathogenic variants in *BRCA* and other breast cancer risk genes were detected less frequently in Aboriginal women in this cohort. However, as most women were not tested, the current data cannot establish whether this reflects a genuinely lower population level prevalence of pathogenic variants or differences in referral, selection, and testing practices. Therefore, further research is warranted to explore the possible presence of pathogenic variants that may be private to Aboriginal women with breast cancer. Developing an Aboriginal-specific reference for cancer risk genes is logistically and financially challenging but important to allow for the inclusion of ancestry-specific variants in routine genetic testing panels for at-risk individuals. This in turn will enhance equity, making advances in personalized care available to all those affected and their families.

## Figures and Tables

**Table 1:** Demographics of Aboriginal and non-Aboriginal cohorts.

	Aboriginal *n* = 259		Non-Aboriginal *n* = 789		Significance (Chi-Square)
Age at Diagnosis					
Range	24–98		24–97		*p* = 0.86 *
Mean (years)	55.8		55.5		
Median	55		55		
Residence (ARIA)	*n*	%	*n*	%	
Major cities	89	(34.4)	274	(34.7)	ARIA5 *p* < 0.001
Inner regional	8	(3.1)	35	(4.4)	
Outer regional	32	(12.4)	114	(14.3)	
Remote	67	(25.9)	293	(37.3)	ARIA3 *p* = 0.49
Very remote	63	(24.3)	73	(9.3)	
SE disadvantage (IRSD)					
1 (most disadvantaged)	101	(39.0)	104	(13.2)	*p* < 0.001
2	66	(25.5)	186	(23.6)	
3	51	(19.7)	197	(25.0)	
4	18	(6.9)	144	(18.3)	
5 (least disadvantaged)	23	(8.9)	158	(20.0)	
Year of Diagnosis					
up to 2003	44	(17.0)	128	(16.2)	*p* = 0.51
2004–2006	26	(10.0)	104	(13.2)	
2007–2009	42	(16.2)	99	(12.5)	
2010–2012	67	(25.9)	201	(25.5)	
2013 onwards	80	(30.9)	257	(32.6)	

* *t*-test significance test.

**Table 2:** Tumour features in Aboriginal and non-Aboriginal cohorts.

		Aboriginal		Non-Aboriginal		Significance	OR	95% CI	*p*
		*n*	%	*n*	%	(Chi-sq)			
Tumour Staging									
Primary Size	Tx *	41	(15.8)	115	(14.6)	<0.001	1.87	1.36–2.59	<0.001
	T0/1	112	(43.2)	448	(56.8)				
	T2	94	(36.3)	192	(24.3)				
	T3	10	(3.9)	33	(4.2)				
	T4	2	(0.8)	1	(0.1)				
	Total	259	(100.0)	789	(100.0)				
Lymph Nodes	Nx *	26	(10.0)	71	(9.0)		1.25	0.91–1.71	0.158
	N0	140	(54.1)	469	(59.4)				
	N1	62	(23.9)	169	(21.4)				
	N2/3	31	(12.0)	80	(10.1)				
	Total	259	(100.0)	789	(100.0)				
Metastasis	Mx *	2	(0.8)	7	(0.9)		1.69	0.93–3.02	0.065
	M0	236	(91.1)	743	(94.2)				
	M1	21	(8.1)	39	(4.9)				
	Total	259	(100.0)	789	(100.0)				
Tumour Biology									
Invasive Grade	unknown *	13	(5.0)	37	(4.7)	<0.001	1.79	1.32–2.43	<0.001
	1	33	(12.7)	183	(23.2)				
	2	103	(39.8)	335	(42.5)				
	3	110	(42.5)	234	(29.7)				
	Total	259	(100.0)	789	(100.0)				
Oestrogen Receptor	unknown *	6	(2.3)	33	(4.2)	0.016	1.49	1.05–2.10	0.021
	Positive	186	(71.8)	609	(77.2)				
	Negative	67	(25.9)	147	(18.6)				
	Total	259	(100.0)	789	(100.0)				
Progesterone Receptor	unknown *	6	(2.3)	36	(4.6)	0.027	1.42	1.03–1.95	0.030
	Positive	166	(64.1)	550	(69.7)				
	Negative	87	(33.6)	203	(25.7)				
	Total	259	(100.0)	789	(100.0)				
Triple Negative	No	205	(84.0)	621	(87.6)		1.34	0.90–1.98	0.157
	Yes	39	(16.0)	88	(12.4)				
	Total	244	100	709	100				
HER2 Receptor	Positive	52	(21.3)	95	(13.3)		1.76	1.18–2.59	0.004
	Negative	192	(78.7)	617	(86.7)				
	Total	244	100	712	100				

* ‘x’ data not available. Where staging categories were combined into two groups for effect size estimation (Primary Size: T0/1 vs. T2–T4; Invasive Grade: Grade 3 vs. Grade 1–2), the *p*-value in the OR/CI row reflects the two-group comparison, which may differ slightly from the omnibus chi-square *p*-value for the full multi-category comparison shown in the Significance column.

**Table 3:** Family history of breast and other cancers by Aboriginal status.

	Aboriginal		Non-Aboriginal		Significance (Chi-Square)
	*n*	%	*n*	%	
Total Cohort	259	(100.0)	789	(100.0)	
FHx data available	120	(46.3)	268	(34.0)	*p* < 0.001, OR 1.68 CI 1.25–2.25
Any FHx Ca	59	(49.2)	149	(55.6)	0.24, OR 0.77 CI 0.49–1.22
FHx *BRCA* or OvCa	53	(44.2)	138	(51.5)	0.18, OR 0.75 CI 0.47–1.17
First-degree FHx *BRCA*	27	(22.5)	60	(22.4)	0.98, OR 1.01 CI 0.58–1.73
Second-degree FHx *BRCA*	23	(19.2)	64	(23.9)	0.30, OR 0.76 CI 0.42–1.32
First/second-degree FHx *BRCA*	45	(37.5)	106	(39.6)	0.70, OR 0.92 CI 0.57–1.46
Any FHx *BRCA*	51	(42.5)	124	(46.3)	0.49, OR 0.86 CI 0.54–1.35
First-degree FHx OvCa	5	(4.2)	6	(2.2)	0.29, OR 1.90 CI 0.45–7.62
Second-degree FHx OvCa	1	(0.8)	10	(3.7)	0.11, OR 0.22 CI 0.00–1.56
First/second-degree FHx OvCa	6	(5.0)	16	(6.0)	0.70, OR 0.83 CI 0.26–2.30
Any FHx OvCa	8	(6.7)	17	(6.3)	0.90, OR 1.05 CI 0.38–2.67
First-degree FHx Endometrial Ca	1	(0.8)	2	(0.7)	0.93 OR 1.12 CI 0.02–21.66
Second-degree FHx Endometrial Ca	0	(0.0)	0	(0.0)	-
First/second-degree FHx Endometrial Ca	1	(0.8)	2	(0.8)	-
Any FHx Endometrial Ca	3	(2.5)	2	(0.7)	0.16 OR 3.40 CI 0.38–41.23

All percentages are calculated among patients with family history data available (Aboriginal *n* = 120, non-Aboriginal *n* = 268), except “FHx data available”, which is calculated from the full cohort (Aboriginal *n* = 259, non-Aboriginal *n* = 789). Fisher’s exact test was used for <5 counts; chi-square testing was used otherwise. Note: the adjusted odds ratio for family history data availability (Section 3.2, adjusted for IRSD decile and tumour grade) is reported in the text; the OR/CI shown in this table is the unadjusted (crude) estimate. Family history (FHx), cancer (Ca), breast cancer (*BRCA*), ovarian cancer (OvCa).

**Table 4:** Germline testing by Aboriginal status.

	Aboriginal		Non-Aboriginal		Significance
	*n*	(%)	*n*	(%)	
Total Cohort	259	(100.0)	789	(100.0)	
Assessed for eligibility	14	(5.4)	57	(7.2)	0.392, OR 0.73 CI 0.37–1.36
Offered testing	12	(4.6)	46	(5.8)	0.533, OR 0.78 CI 0.37–1.54
Accepted testing	10	(3.9)	44	(5.6)	0.333, OR 0.68 CI 0.30–1.40
Detection in full cohort					
Any *BRCA* variant	2	(0.77)	19	(2.4)	0.127, OR 0.32 CI 0.04–1.32
Any pathogenic variant	2	(0.77)	24	(3.0)	0.039, OR 0.25 CI 0.028–1.01
Positivity among those tested	10	(3.9)	44	(5.6)	
Any *BRCA* variant	2	(20.0)	19	(43.2)	0.284, OR 0.34 CI 0.03–1.96
Any pathogenic variant	2	(20.0)	24	(54.5)	0.079, OR 0.21 CI 0.020–1.24
Negative/inconclusive/unknown significance	8	(80.0)	20	(45.5)	0.079

Detection rates represent identified variants across the full cohort regardless of testing status. Positivity rates represent outcomes among patients who underwent germline testing only. Fisher’s exact test was used for all comparisons. Note that adjusted odds ratios for referral and acceptance of testing (Section 3.3, adjusted for IRSD decile and tumour grade) are reported in the text rather than this table, as they derive from multivariable models rather than the pairwise comparisons shown here.

## Data Availability

The data for this study will not be shared, as we do not have permission from the participants or ethics approval to do so.

## Abbreviations

The following abbreviations are used in this manuscript:

*BRCA*1	BReast CAncer gene 1
*BRCA*2	BReast CAncer gene 2
WACR	Western Australian Cancer Registry
ARIA	Accessibility/Remoteness Index of Australia)
IRSD	Index of Relative Socioeconomic Disadvantage
PARP	Poly (ADP-ribose) polymerase
WAAHEC	WA Aboriginal Human Ethics Committee
FHx	Family history
Ca	Cancer
*BRCA*	Breast cancer
OvCa	Ovarian cancer

## References

[1] Tapia K.A., Garvey G., Mc Entee M., Rickard M., Brennan P. (2017). Breast Cancer in Australian Indigenous Women: Incidence, Mortality, and Risk Factors. Asian Pac. J. Cancer Prev..

[2] AIHW (2018). Cancer in Aboriginal & Torres Strait Islander People of Australia.

[3] Cunningham J., Rumbold A.R., Zhang X., Condon J.R. (2008). Incidence, aetiology, and outcomes of cancer in Indigenous peoples in Australia. Lancet Oncol..

[4] Nicolis O., De Los Angeles D., Taramasco C. (2024). A contemporary review of breast cancer risk factors and the role of artificial intelligence. Front. Oncol..

[5] Mbuya-Bienge C., Pashayan N., Kazemali C.D., Lapointe J., Simard J., Nabi H. (2023). A Systematic Review and Critical Assessment of Breast Cancer Risk Prediction Tools Incorporating a Polygenic Risk Score for the General Population. Cancers.

[6] Gasparri M.L., Bellaminutti S., Farooqi A.A., Cuccu I., Di Donato V., Papadia A. (2022). Endometrial Cancer and *BRCA* Mutations: A Systematic Review. J. Clin. Med..

[7] Keane F., O’Connor C.A., Park W., Seufferlein T., O’Reilly E.M. (2023). Pancreatic Cancer: *BRCA* Targeted Therapy and Beyond. Cancers.

[8] Maccaroni E., Giampieri R., Lenci E., Scortichini L., Bianchi F., Belvederesi L., Brugiati C., Pagliaretta S., Ambrosini E., Berardi R. (2021). *BRCA* mutations and gastrointestinal cancers: When to expect the unexpected?. World J. Clin. Oncol..

[9] Antoniou A., Pharoah P.D., Narod S., Risch H.A., Eyfjord J.E., Hopper J.L., Loman N., Olsson H., Johannsson O., Borg A. (2003). Average risks of breast and ovarian cancer associated with *BRCA1* or *BRCA2* mutations detected in case Series unselected for family history: A combined analysis of 22 studies. Am. J. Hum. Genet..

[10] Yoshida R. (2021). Hereditary breast and ovarian cancer (HBOC): Review of its molecular characteristics, screening, treatment, and prognosis. Breast Cancer.

[11] Anglian Breast Cancer Study Group (2000). Prevalence and penetrance of *BRCA1* and *BRCA2* mutations in a population-based series of breast cancer cases. Br. J. Cancer..

[12] Domchek S.M., Yao S., Chen F., Hu C., Hart S.N., Goldgar D.E., Nathanson K.L., Ambrosone C.B., Haiman C.A., Couch F.J. (2021). Comparison of the Prevalence of Pathogenic Variants in Cancer Susceptibility Genes in Black Women and Non-Hispanic White Women with Breast Cancer in the United States. JAMA Oncol..

[13] Ding R., Xiao Y., Mo M., Zheng Y., Jiang Y.Z., Shao Z.M. (2022). Breast cancer screening and early diagnosis in Chinese women. Cancer Biol. Med..

[14] Lattimore V., Parsons M.T., Spurdle A.B., Pearson J., Lehnert K., Sullivan J., Lintott C., Bawden S., Morrin H., Robinson B. (2021). Under-ascertainment of breast cancer susceptibility gene carriers in a cohort of New Zealand female breast cancer patients. Breast Cancer Res. Treat..

[15] Liede A., Jack E., Hegele R.A., Narod S.A. (2002). A BRCA1 mutation in Native North American families. Hum. Mutat..

[16] Elston C.W., Ellis I.O. (1991). Pathological prognostic factors in breast cancer. I. The value of histological grade in breast cancer: Experience from a large study with long-term follow-up. Histopathology.

[17] National Health and Medical Research Council (2018). Ethical Conduct in Research with Aboriginal and Torres Strait Islander Peoples and Communities: Guidelines for Researchers and Stakeholders.

[18] Geyer C.E., Garber J.E., Gelber R.D., Yothers G., Taboada M., Ross L., Rastogi P., Cui K., Arahmani A., Aktan G. (2022). Overall survival in the OlympiA phase III trial of adjuvant olaparib in patients with germline pathogenic variants in *BRCA1/2* and high-risk, early breast cancer. Ann. Oncol..

[19] Robson M.E., Im S.A., Senkus E., Xu B., Domchek S.M., Masuda N., Delaloge S., Tung N., Armstrong A., Dymond M. (2023). OlympiAD extended follow-up for overall survival and safety: Olaparib versus chemotherapy treatment of physician’s choice in patients with a germline *BRCA* mutation and HER2-negative metastatic breast cancer. Eur. J. Cancer.

[20] Southey M.C., Dowty J.G., Riaz M., Steen J.A., Renault A.-L., Tucker K., Kirk J., James P., Winship I., Pachter N. (2021). Population-based estimates of breast cancer risk for carriers of pathogenic variants identified by gene-panel testing. npj Breast Cancer.

[21] Rebbeck T.R., Friebel T.M., Friedman E., Hamann U., Huo D., Kwong A., Olah E., Olopade O.I., Solano A.R., Teo S.-H. (2018). Mutational spectrum in a worldwide study of 29,700 families with *BRCA1* or *BRCA2* mutations. Hum. Mutat..

[22] Wang S.M. (2023). A global perspective on the ethnic-specific *BRCA* variation and its implication in clinical application. J. Natl. Cancer Cent..

[23] Qin Z., Huang T., Guo M., Wang S.M. (2022). Distinct landscapes of deleterious variants in DNA damage repair system in ethnic human populations. Life Sci. Alliance.

[24] Parmigiani G., Berry D., Aguilar O. (1998). Determining carrier probabilities for breast cancer-susceptibility genes *BRCA1* and *BRCA2*. Am. J. Hum. Genet..

[25] Frank T.S., Deffenbaugh A.M., Reid J.E., Hulick M., Ward B.E., Lingenfelter B., Gumpper K.L., Scholl T., Tavtigian S.V., Pruss D.R. (2002). Clinical characteristics of individuals with germline mutations in *BRCA1* and *BRCA2*: Analysis of 10,000 individuals. J. Clin. Oncol..

[26] Kurian A.W., Gong G.D., Chun N.M., Mills M.A., Staton A.D., Kingham K.E., Crawford B.B., Lee R., Chan S., Donlon S.S. (2008). Performance of *BRCA1/2* mutation prediction models in Asian Americans. J. Clin. Oncol..

[27] Kang E., Kim S.-W. (2013). The Korean Hereditary Breast Cancer Study: Review and Future Perspectives. J. Breast Cancer.

[28] De Silva D.L., Stafford L., Skandarajah A.R., Sinclair M., Devereux L., Hogg K., Kentwell M., Park A., Lal L., Zethoven M. (2023). Universal genetic testing for women with newly diagnosed breast cancer in the context of multidisciplinary team care. Med. J. Aust..

[29] Lopez A., Nichols Doyle R., Sandoval C., Nisson K., Yang V., Fregoso O.I. (2022). Viral Modulation of the DNA Damage Response and Innate Immunity: Two Sides of the Same Coin. J. Mol. Biol..

[30] Zhao B., Li J., Sinha S., Qin Z., Kou S.H., Xiao F., Lei H., Chen T., Cao W., Ding X. (2024). Pathogenic variants in human DNA damage repair genes mostly arose in recent human history. BMC Cancer.

[31] Bernardes C.M., Valery P.C., Garvey G. (2014). Exploring the cancer risk perception and interest in genetic services among Indigenous people in Queensland, Australia. Aust. N. Z. J. Public Health.

[32] Tobler R., Rohrlach A., Soubrier J., Bover P., Llamas B., Tuke J., Bean N., Abdullah-Highfold A., Agius S., O’dOnoghue A. (2017). Aboriginal mitogenomes reveal 50,000 years of regionalism in Australia. Nature.

[33] Cancer C. (2001). Familial breast cancer: Collaborative reanalysis of individual data from 52 epidemiological studies including 58,209 women with breast cancer and 101,986 women without the disease. Lancet.

[34] Taylor R., Boyages J. (2000). Absolute risk of breast cancer for Australian women with a family history. Aust. N. Z. J. Surg..

[35] ABS (2022). Western Australia: Aboriginal and Torres Strait Islander Population Summary.

[36] Palmer J.R., Polley E.C., Hu C., John E.M., Haiman C., Hart S.N., Gaudet M., Pal T., Anton-Culver H., Trentham-Dietz A. (2020). Contribution of Germline Predisposition Gene Mutations to Breast Cancer Risk in African American Women. J. Natl. Cancer Inst..

[37] Cragun D., Weidner A., Lewis C., Bonner D., Kim J., Vadaparampil S.T., Pal T. (2017). Racial disparities in BRCA testing and cancer risk management across a population-based sample of young breast cancer survivors. Cancer.

[38] Jacobs B., Roffenbender J., Collmann J., Cherry K., Bitsói L.L., Bassett K., Evans C.H. (2010). Bridging the divide between genomic science and Indigenous peoples. J. Law. Med. Ethics.

[39] Kowal E., Gallacher L., Macciocca I., Sahhar M. (2015). Genetic Counseling for Indigenous Australians: An Exploratory Study from the Perspective of Genetic Health Professionals. J. Genet. Couns..

[40] Aboriginal Studies Press (2020). AIATSIS Code of Ethics for Aboriginal and Torres Strait Islander Research.

[41] United Nations (2007). United Nations Declaration on the Rights of Indigenous Peoples.

[42] Carroll S.R., Garba I., Figueroa-Rodríguez O.L., Holbrook J., Lovett R., Materechera S., Parsons M., Raseroka K., Rodriguez-Lonebear D., Rowe R. (2020). The CARE Principles for Indigenous Data Governance. Data Sci. J..

[43] Wilkinson M.D., Dumontier M., Aalbersberg I.J., Appleton G., Axton M., Baak A., Blomberg N., Boiten J.W., da Silva Santos L.B., Bourne P.E. (2016). The FAIR Guiding Principles for scientific data management and stewardship. Sci. Data.

[44] Dossa F., Metcalfe K., Sutradhar R., Little T., Eisen A., Chun K., Meschino W.S., Velsher L., Ellis J.L., Baxter N.N. (2021). Building the What Comes Next Cohort for *BRCA1* and *BRCA2* testing: A descriptive analysis. CMAJ Open.

[45] Lammert J., Skandarajah A.R., Shackleton K., Calder P., Thomas S., Lindeman G.J., Mann G.B. (2020). Outcomes of women at high familial risk for breast cancer: An 8-year single-center experience. Asia Pac. J. Clin. Oncol..

[46] Kowal E., Pearson G., Rouhani L., Peacock C.S., Jamieson S.E., Blackwell J.M. (2012). Genetic research and aboriginal and Torres Strait Islander Australians. J. Bioeth. Inq..

[47] Malone K.E., Daling J.R., Neal C., Suter N.M., O’Brien C., Cushing-Haugen K., Jonasdottir T.J., Thompson J.D., Ostrander E.A. (2000). Frequency of *BRCA1*/*BRCA2* mutations in a population-based sample of young breast carcinoma cases. Cancer.

[48] Greenwood H.I., Dodelzon K. (2024). Screening in Women with *BRCA* Mutations Revisited. J. Breast Imaging.

[49] Kotsopoulos J., Gronwald J., Huzarski T., Aeilts A., Randall Armel S., Karlan B., Singer C.F., Eisen A., Tung N., Olopade O. (2023). Tamoxifen and the risk of breast cancer in women with a *BRCA1* or *BRCA2* mutation. Breast Cancer Res. Treat..

[50] Nemati Shafaee M., Goutsouliak K., Lin H., Bevers T.B., Gutierrez-Barrera A., Bondy M., Arun B. (2022). Aromatase inhibitors and contralateral breast cancer in *BRCA* mutation carriers. Breast Cancer Res. Treat..

[51] Tutt A., Tovey H., Cheang M.C.U., Kernaghan S., Kilburn L., Gazinska P., Owen J., Abraham J., Barrett S., Barrett-Lee P. (2018). Carboplatin in *BRCA1/2*-mutated and triple-negative breast cancer BRCAness subgroups: The TNT Trial. Nat. Med..

